# Rbfox1 expression in amacrine cells is restricted to GABAergic and VGlut3 glycinergic cells

**DOI:** 10.1042/BSR20220497

**Published:** 2022-07-08

**Authors:** Lei Gu, Joseph Caprioli, Natik Piri

**Affiliations:** 1 Stein Eye Institute, University of California, Los Angeles, Los Angeles, CA 90095, U.S.A.; 2 Brain Research Institute, University of California, Los Angeles, Los Angeles, CA 90095, U.S.A.

**Keywords:** amacrine cells, Choline acetyltransferase, ganglion cells, glycine transporter, Vesicular glutamate transporter 3

## Abstract

Rbfox1 is a multifunctional RNA-binding protein that regulates alternative splicing, transcription, mRNA stability, and translation. Rbfox1 is an important regulator of gene networks involved in neurogenesis and neuronal function. Disruption of Rbfox function has been associated with several neurodevelopmental and neuropsychiatric disorders. We have shown earlier that Rbfox1 is expressed in retinal ganglion and amacrine cells (ACs) and that its down-regulation in adult mouse retinas leads to deficiency of depth perception. In the present study, we used several markers of ACs, including gamma-aminobutyric acid (GABA), choline acetyltransferase (ChAT), neuropeptide Y (NPY), glycine transporter (GlyT1), and vesicular glutamate transporter 3 (VGlut3) to identify types of ACs that express Rbfox1. Expression of Rbfox1 was observed predominantly in GABAergic ACs located in the inner nuclear layer (INL) and ganglion cell layer (GCL). All GABAergic/cholinergic starburst ACs and virtually all NPY-positive GABAergic ACs were also Rbfox1-positive. Among glycinergic ACs, a sparse population of Rbfox1/VGlut3-positive cells was identified, indicating that Rbfox1 is expressed in a very small population of glycinergic ACs. These data contribute to our understanding about molecular differences between various types of amacrine cells and the cell-specific gene networks regulated by Rbfox1.

## Introduction

The members of Rbfox family of RNA-binding protein, Rbfox1, Rbfox2, and Rbfox3, regulate cell- or tissue-specific alternative splicing, miRNA processing, mRNA stability, and translation efficiency [1]. The diverse functions of each Rbfox protein are supported by spatiotemporal expression of distinct isoforms from different promoters or by alternative splicing [1,2]. These proteins contain a single highly conserved RNA recognition motif (RRM)-type RNA-binding domain that facilitates its binding to (U)GCAUG element at various regulatory sites within alternatively spliced exons, pre-mRNA introns, pre-miRNAs hairpin structures, and mRNA 3′ untranslated region. Rbfox proteins regulate extensive genetic networks in both developing and mature tissues; mutations, chromosomal translocation, or deletions leading to aberrations in Rbfox-regulated circuitry have been linked to several neurodevelopmental and neuropsychiatric disorders, sleep latency, and cardiac hypertrophy [3–9].

We first identified Rbfox gene expression in the retina by analyzing the transcriptome of retinal ganglion cells (RGCs) [10]. We recently reported results on the expression patterns of Rbfox1 and Rbfox2 in the retina and changes in the retinal transcriptome and visual function due to the down-regulation of these proteins [11,12]. The key findings of the present work are: in mature mouse retinas, both Rbfox1 and Rbfox2 were expressed in all types of RGCs and certain types of amacrine cells (ACs); Rbfox2 was present in a wider population of ACs than Rbfox1 in both the inner nuclear layer (INL) and ganglion cell layer (GCL); Rbfox2 but not Rbfox1 was also expressed in horizontal cells; Rbfox1 expression shifted from cytoplasmic to predominantly nuclear at around P0 and remained so in mature retinas, whereas Rbfox2 localization was predominantly nuclear during retinogenesis and in adult retinas; deletion of Rbfox1 and Rbfox2 in adult animals had no detectable effect on retinal morphology, but the both Rbfox1 and Rbfox2 knockout animals had deficient depth perception; retinal transcriptome analysis identified a number of Rbfox1/Rbfox2-regulated genes associated with synaptic functions and Rbfox2-regulated genes associated with circadian rhythm/entrainment pathways.

In the present study, we characterize the expression of Rbfox1 in different types of ACs. ACs are inhibitory interneurons; they receive excitatory inputs from bipolar cells, make feedback synapses onto bipolar cells, feedforward synapses onto RGCs, and lateral inhibitory synapses onto other ACs [13]. Their activity, interactions, and rich diversity underlie complex, spatiotemporal processing of visual information encoded by RGCs. The diversity of ACs was suggested to correlate with the diversity of RGCs [14]. Recent study on molecular characterization of ACs with high-throughput single-cell RNA sequencing identified 63 AC types in the mouse retina [15]. Functional characterization based on the type of primary neurotransmitter expression classifies these cells into two groups: wide-field gamma-aminobutyric acid (GABA)-ergic and narrow-field glycinergic ACs [16,17]. Out of 63 types of ACs identified with single-cell sequencing, 43 types were GABAergic and 13 types were glycinergic [15]. GABAergic and glycinergic cells comprise approximately 35 and 40% of all ACs, respectively [18]. In addition to ACs that express these canonical inhibitory neurotransmitters, there are AC types that express neither or both, or express other neuropeptides or neuromodulators. In the present study, we have used several AC-specific markers, including GABA, choline acetyltransferase (ChAT), neuropeptide Y (NPY), glycine transporter (GlyT1) and vesicular glutamate transporter 3 (VGlut3) to identify types of ACs that express Rbfox1.

## Methods

### Animals

All experiments with animals were approved by the UCLA institutional animal care and use committee (IACUC) that oversee the use of animals in research, teaching, and testing to ensure the humane treatment and proper care of animals (protocol # ARC-2017-099; title Rbfox1 and Rbfox2 in retinal development and function; PI Natik Piri; approval date 10/25/2021; expiration date 10/24/2024) and were performed in compliance with the National Institutes of Health Guide for the Care and Use of Animals and the Association for Research in Vision and Ophthalmology Statement for the Use of Animals in Ophthalmic and Vision Research. Male and female wild-type C57BL/6J mice were used in the present study. Mice were housed in the UCLA Center for the Health Sciences (CHS) vivarium in a room with an ambient temperature of 25°C, 30–70% humidity, a standard 12/12-h light/dark cycle, with food and water provided *ad libitum*. Animals were euthanized by CO_2_ asphyxiation for postmortem tissue collection (location: UCLA Jules Stein Eye Institute, Room BH-778).

### Immunohistochemistry

The primary antibodies for Rbfox1, Rbpms, calbindin, GABA, ChAT, NPY, and VGlut3 were used for immunohistochemistry to identify retinal cells that express Rbfox1. These antibodies are highly specific for their targets and have been extensively used in the field [11,15,19–23]. Detailed information about primary and secondary antibodies is presented in Table 1. Immunohistochemistry was performed following a standard procedure with minor modifications. In brief, eyes were enucleated, fixed with ice-cold 4% paraformaldehyde, and cryoprotected in 30% sucrose. The 14-µm thick retinal sections were cut with cryostat. Sections were incubated with blocking solution (20% fetal calf serum, 5% goat serum, 0.1% Triton X-100 in phosphate buffered saline (PBS)) for 30 min followed by incubation with primary antibodies overnight at 4°C. After washing three times with 0.1% Triton X-100 in PBS, sections were incubated with secondary antibodies for 1 h at room temperature, washed again three times, and mounted with mounting medium containing DAPI (4′,6-diamidino-2-phenylindole; Sigma-Aldrich, St. Louis, MO, U.S.A.) reagent for nuclear staining. Immunolabeled retinal sections were imaged with a confocal laser scanning microscope Olympus FV3000 (Olympus, MA, U.S.A.). For quantitative analysis, cells in the INL and GCL immunostained with AC marker and with AC markers/Rbfox1 were counted and presented as a mean ± standard deviation per retinal section. Each experiment included retinas from at least three animals.

**Table 1:** Primary and secondary antibodies used for immunohistochemistry

Antibody	Manufacturer	Catalog #	Type	Host	Dilution
**Primary antibodies**					
Calbindin	EMD Millipore	PC253L	Polyclonal	Rabbit	1:500
ChAT	EMD Millipore	AB144P	Polyclonal	Goat	1:200
GABA	Sigma	A2052	Polyclonal	Rabbit	1:2000
GlyT1	Thermo Fisher	PA5-53103	Polyclonal	Guinea pig	1:100
NPY	Abcam	ab221145	Monoclonal	Rabbit	1:1000
vGlut3	EMD Millipore	AB5421	Polyclonal	Guinea pig	1:2500
Rbfox1	Novus Biologicals	NBP2-13169	Monoclonal	Mouse	1:200
Rbpms	PhosphoSolutions	1832-RBPMS	Polyclonal	Guinea pig	1:1000
**Secondary antibodies** (Thermo Fisher)					
Alexa Fluor 488 antirabbit		A-21206	Polyclonal	Donkey	1:500
Alexa Fluor 488 antigoat		A-11055	Polyclonal	Donkey	1:500
Alexa Fluor 488 antiguinea pig		A-11073	Polyclonal	Goat	1:500
Alexa Fluor 568 antimouse		A-10037	Polyclonal	Donkey	1:500

## Results and discussion

### Expression of Rbfox1 in mouse retinas is restricted to RGCs and ACs

**Figure 1:**
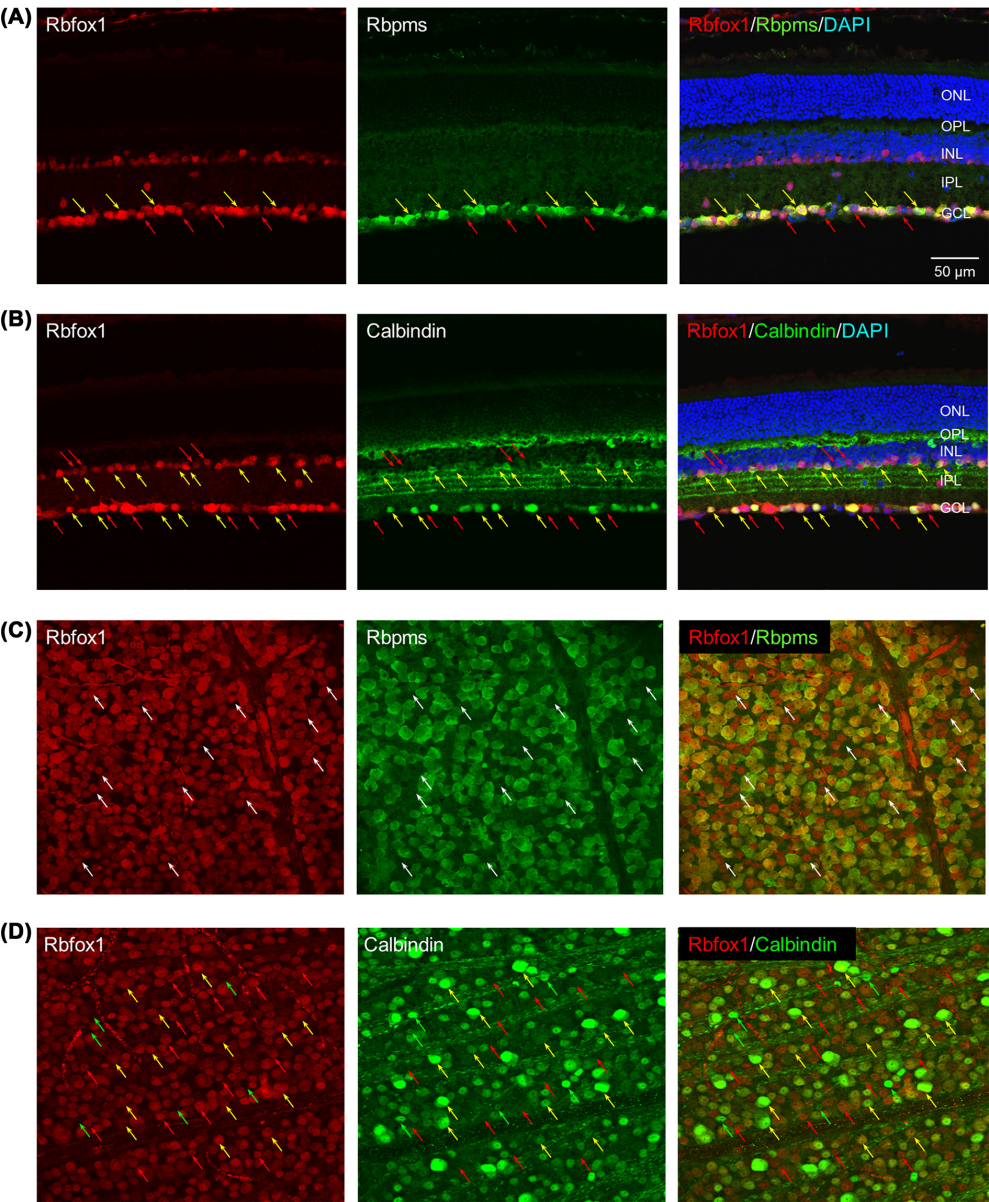
Rbfox1 is expressed in RGCs and ACs (**A**) In the GCL, all Rbpms-positive cells, RGCs, were also stained for Rbfox1 (yellow arrows point at some Rbpms/Rbfox1-positive cells). Red arrows point at some Rbfox1-positive/Rbpms-negative cells that are most likely displaced ACs. (**B**) In the INL, virtually all clabindin-positive cells were also Rbfox1-positive cells (yellow arrows). Very few Rbfox1-immunostained cells appeared to be calbindin-negative (red arrows). In the GCL, Rbfox1 is expressed in calbindin-positive displaced ACs (yellow arrows), as well as in calbindin-negative cells, many of which are RGCs (red arrows). (**C**) Colocalization of Rbfox1 expression with RGCs in whole mount retinas. All Rbpms-positive cells were immunostained with Rbfof1. Rbfox1-positive/Rbpms-negative cells (presumably displaced ACs) are indicated by white arrows. (**D**) A significant overlap in Rbfox1 and calbindin expression within GCL was observed in whole mount retinas (yellow arrows). Rbfox1-positive/calbindin-negative and calbindin-positive/Rbfox1-negative cells are pointed with red and green arrows, respectively. DAPI; 4',6-diamidino-2-phenylindole; GCL; ganglion cell layer, INL; inner nuclear layer, IPL; inner plexiform layer, ONL; outer nuclear layer, OPL; outer plexiform layer.

Rbfox1 spatial expression in the retina was evaluated by immunohistochemistry with anti-Rbfox1 antibodies. Rbfox1-positive cells were located in the INL, which contains cell bodies of horizontal cells, bipolar cells, and ACs, as well as Muller glial cells and in GCL, which in rodent retinas contain RGCs and displaced ACs, in approximately 1:1 ratio (in mouse retinas, there are ∼45% of RGCs and ∼55% of dACs; Figure 1A,B) [22]. The vast majority of cells in the GCL were Rbfox1-positive, implying that both RGCs and dACs express Rbfox1. In the INL, however, Rbfox1 was expressed only in cells adjacent to the inner plexiform layer (IPL). Since the innermost two to four rows of cells in the mouse retinal INL are known to be formed by ACs [21,24], these Rbfox1-positive cells are most likely to be ACs. To demonstrate that the observed Rbfox1-positive cells are RGCs and ACs, double immunostaining with Rbfox1 and Rbpms or calbindin, RGC, and AC markers, respectively, was performed. In the GCL, practically all Rbpms-labeled cells were also positive for Rbfox1 (Figure 1A; pointed with yellow arrows). Rbfox1-positive/Rbpms-negative cells in the GCL are in all probability dACs (Figure 1A, red arrows).

Expression of Rbfox1 in ACs was analyzed in retinal sections and whole mounts by coimmunolabeling with antibodies against Rbfox1 and calcium-binding protein calbindin-D28K. Calcium-binding proteins—∼250 calcium-binding proteins have been identified in mice [25]—participate in calcium-mediated signaling pathways that regulate many cellular processes. In the central nervous system, the most well-described calcium-binding proteins include parvalbumin (PV), calbindin-D28K (CB), calretinin (CR), calmodulin (CM), calcineurin, and the S100 family. These proteins are commonly used as immunohistochemical markers for discrete neuronal subpopulations [26]. Immunohistochemistry of mouse retinal sections for calbindin showed expression of this protein in cells within INL and GCL (Figure 1B). Furthermore, calbindin immunostaining was observed in the outer plexiform layer (OPL) as well as in the IPL, in which the staining pattern appears as three distinct bands. The inner and outer bands in this three-layered stratification of IPL are colocalized with the fibers of cholinergic AC/dAC (colocalization of Rbfox1 with cholinergic starburst AC is described below), whereas the band in the middle corresponds to the fibers of the WA-S2/3 wide-field AC population [27,28]. The vast majority of the Rbfox1-postive cells in the INL were also calbindin-positive (Figure 1B, yellow arrows). Very few Rbfox1-positive cells appeared to be negative or very weakly stained for calbindin (Figure 1B, red arrows). In contrast, in the GCL, along with Rbfox1/calbindin-positive cells, we see a significant number of Rbfox1-positive/calbindin-negative cells (Figure 1B,C). It has been shown that in the GCL, calbindin is expressed in both dACs and RGCs: approximately 8% of calbindin-immunoreactive cells are displaced ACs and 92% are RGCs [29]. Calbindin expression in RGCs is restricted to ON–OFF directionally selective RGCs and to sustained ON-α RGCs. In total, approximately 18% of mouse RGCs express calbindin. Therefore, Rbfox1-positive/calbindin-negative cells could be either RGCs or dACs (Figure 1B,D, red arrows). With respect to a relatively small population of Rbfox1-negative/calbindin-positive cells, we think that these cells represent dACs subtype(s) (Figure 1C, green arrows). This suggestion is based on our quantitative analysis of Rbfox1 expression in the GCL that was performed by counting Rbfox1/Rbpms and Rbfox1/calbindin-positive cells in the whole-mounted retinas [11]. Virtually 100% of Rbpms-positive cells (RGCs) were also positive for Rbfox1 (3644 ± 104.44 cells/mm^2^), whereas Rbfox1/calbindin-positive cells (2358 ± 47 cells/mm^2^) constituted approximately 94% of cells immunostained with calbindin (2513 ± 46 cells/mm^2^).

### Rbfox1 expression in glycinergic ACs

**Figure 2:**
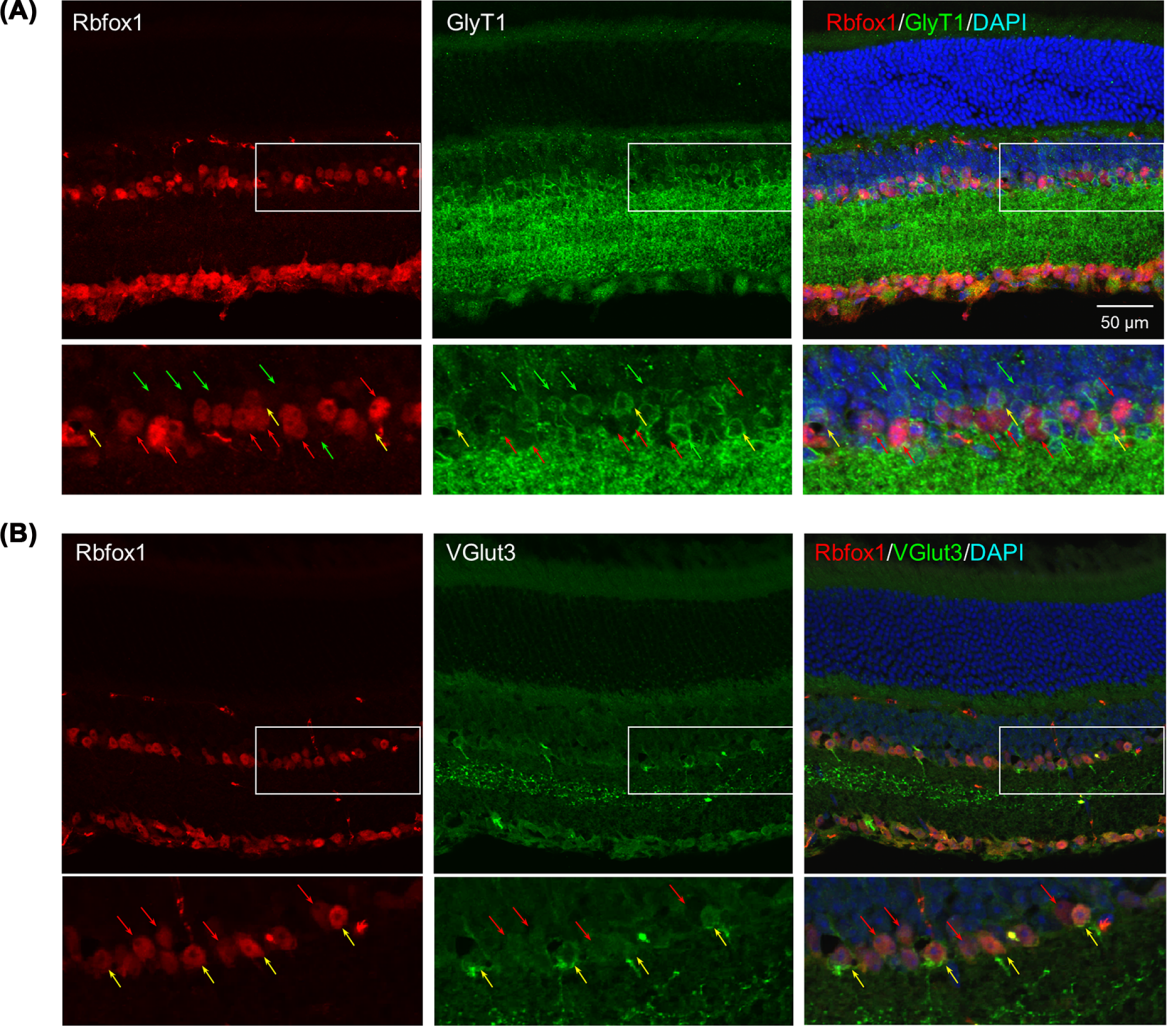
Expression of Rbfox1 in glycinergic ACs (**A**) Rbfox1 is expressed in very few GlyT1-positve ACs (yellow arrows). Rbfox1-positive/GlyT1-negative and GlyT1-positive/Rbfox1-negative cells are pointed with red and green arrows, respectively. (**B**) Rbfox1 is expressed in sparse population of VGlut3-positve glycinergic ACs (yellow arrows). Red arrows point at Rbfox1-positive cells that not immunostained for GlyT1 (A) or for VGlut3 (B). The boxed areas of the vertical retinal sections are shown at higher magnification.

The data presented above imply that Rbfox1 expression within the AC population is restricted to certain subtypes. The majority of ACs expresses canonical inhibitory neurotransmitters GABA or glycine [16,17]. Many of these GABAergic and glycinergic ACs also express other neuromodulators such as acetylcholine, dopamine, somatostatin, substance P, NPY, serotonin vasoactive intestinal polypeptide, and endocannabinoids. To identify Rbfox1-postive ACs, we used several AC subtype-specific markers, including GlyT1, GABA, ChAT, NPY, and VGlut3, for double immunostaining of mouse retinal sections. First, we immunostained retinas with antibodies specific for GlyT1 and Rbfox1 to determine whether Rbfox1 is expressed in glycinergic ACs. It has to be noted that although several cell types in the INL contain glycine, only ACs express the GlyT1 [17,30,31]. Consistent with the location of ACs, GlyT1, and Rbfox1 immunoreactivity within INL was confined to 2–3 rows cells directly adjacent to the IPL (Figure 2A). Colocalization of GlyT1 and Rbfox1 expression showed that the vast majority of glycinergic ACs were Rbfox1 negative and only a sparse population (6–8 cells per retinal section) of GlyT1-positive ACs were stained for Rbfox1 (Figure 1, yellow arrows): out of 79.67 ± 2.05 of GlyT1-positive cells per retinal section, 12 ± 0.82 cells (15%) were GlyT1/Rbfox1 positive. Glycinergic ACs, which constitute 40–50% of all ACs, are represented by more than ten distinct morphological types [17,32–34]. Among them, the best characterized and most numerous type (20–30% of glycinergic ACs in the rat retinas) is the AII ACs [17,32], multistratified neurons with “the most complex interaction repertoire of any known vertebrate neuron” that contribute to both rod-mediated scotopic and cone-mediated photopic vision [35–39]. Another well-studied glycinergic AC type is VGlut3-expressing ACs [40]. VGlut3 cells express both glycine and glutamate. These cells are involved in co-ordinated inhibition and excitation of contrast-suppressed and contrast-enhanced retinal circuits by forming inhibitory glycinergic synapses with uniformity detector RGCs and excitatory glutamatergic synapses with OFF-α and a few other nonlinear contrast-sensitive RGCs [41]. Based on the reported sparse distribution of these cells in the mammalian retinas, we thought that those few Rbfox1-positive glycinergic ACs that we observed in Figure 2A may be VGlut3-expressing cells. To test this possibility, we immunostained retinal sections for Rbfox1 and VGlut3. All VGlut3 ACs in the INL (7 ± 0.82 cells/section) were also positive for Rbfox1, suggesting that within glycinergic AC population, Rbfox1 is expressed predominantly if not exclusively in VGlut3-expressing cells (Figure 2B). Abundant staining for VGlut3 was observed in the GCL. However, we have showed earlier that VGlut3-positive cells in the GCL were also Rbpms-positive, indicating that these cells are RGCs [12].

### Rbfox1 expression in GABAergic ACs

**Figure 3:**
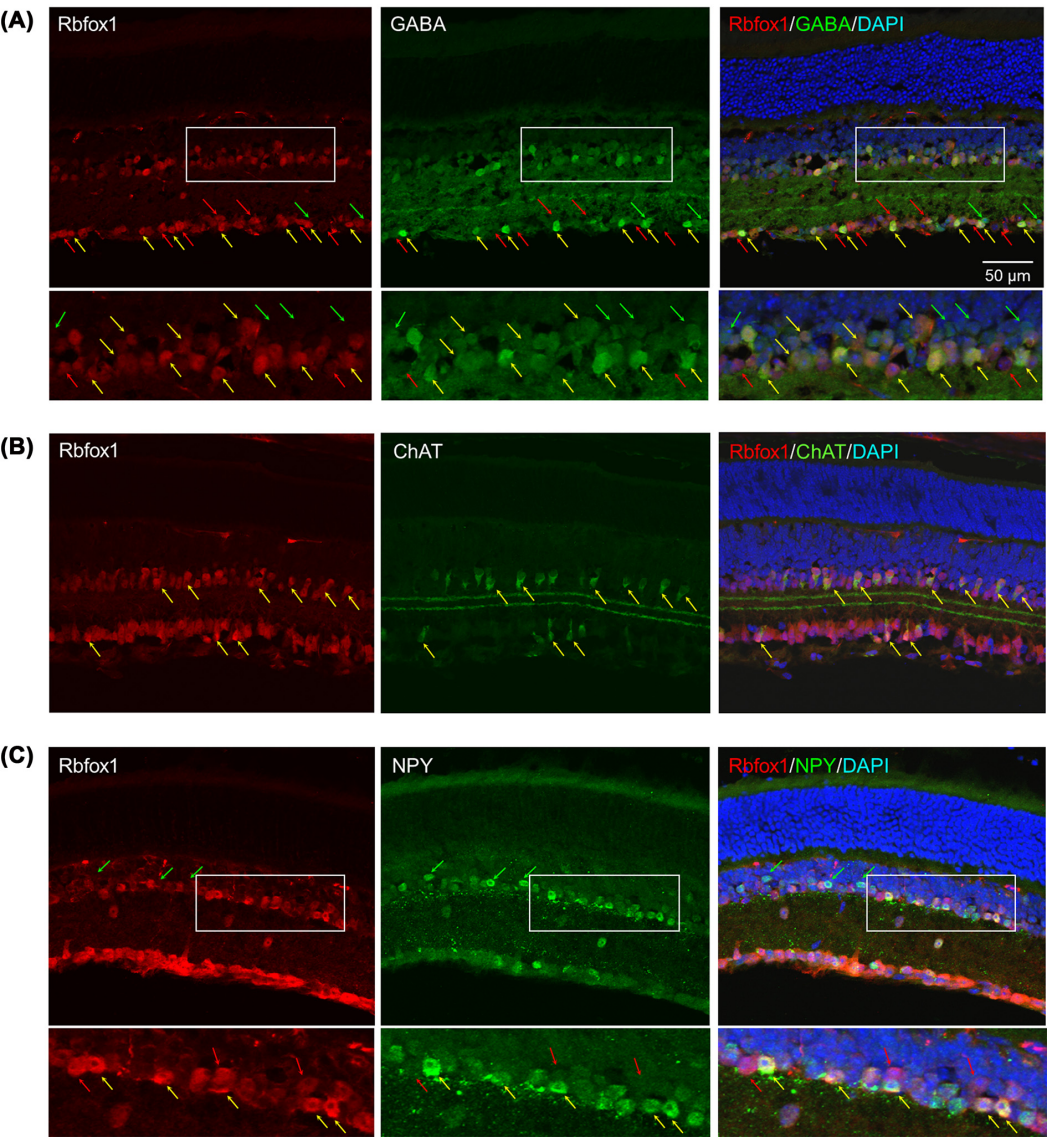
Rbfox1 expression in GABAergic ACs (**A**) The vast majority GABAergic ACs in the INL were Rbfox1-positive. Some GABA/Rbfox2-positive ACs are pointed with yellow arrows. Rbfox1-negative GABAergic ACs and Rbfox1-positve/GABA-negative cells are pointed with green and red arrows, respectively. (**B**) Rbfox1 is expressed in all cholinergic (ChAT-positive) starburst ACs both in the INL and GCL. (**C**) Extensive overlap of Rbfox1 with NPY expression was also observed (yellow arrows). Very few NPY-positive cells had very faint staining for Rbfox1 (pointed by green arrows).

Since only a small number of Rbfox1-positive cells were glycinergic, it was expected that Rbfox1 is predominantly expressed in GABAergic ACs. To characterize Rbfox1 expression in GABAergic ACs, we first immunostained mouse retinal transverse sections with antibodies for Rbfox1 and GABA. GABAergic cells were present both in the GCL (21 ± 0.82 cells/section) and INL (84 ± 1.63 cells/section; Figure 3A). In the GCL, along with Rbfox1/GABA-positive displaced ACs (Figure 3A, yellow arrows), many Rbfox1-positive/GABA-negative cells (Figure 3A, red arrows) were present. Most, if not all, displaced ACs are GABAergic [42] and the fact that very few glycinergic ACs express Rbfox1 suggest that these Rbfox1-positive/GABA-negative cells in the GCL are RGCs. Several GABAergic cells in the GCL appeared to be Rbfox1-negative (Figure 3A, green arrows). However, a faint Rbfox1 staining was also present in those cells. In the INL, a significant overlap in Rbfox1/GABA expression was observed (Figure 3A, yellow arrows). With the exception of 3–6 Rbfox1-positive/GABA-negative cells (most probably glycinergic ACs), all Rbfox1-positive cells adjacent to the IPL were also stained with GABA. This observation is consistent with the above-described data showing that only very few glycinergic ACs express Rbfox1. On the other hand, a population of GABAergic ACs located deeper in the INL showed no Rbfox1 expression (Figure 3A, green arrows). Overall, approximately 87% of GABA-positive cells in the INL were also stained with Rbfox1 (73.33 ± 2.49 cells/section). Next, we used two well-characterized markers, ChAT and NPY, to evaluate Rbfox1 expression in cholinergic and NPY subtypes of GABAergic ACs.

ChAT is the enzyme that catalyzes the biosynthesis of acetylcholine. Its expression in the retina is restricted to starburst ACs. GABAergic/cholinergic starburst ACs regulate direction-selective circuit in the retina by forming cholinergic synapses with On–Off direction-selective RGCs from all directions and GABAergic synapses only from the null direction [43]. Cell bodies of SACs are located in the GCL and INL. Double immunostaining for ChAT and Rbfox1 showed that all starburst ACs in both INL (11.33 ± 0.94 cells/section) and GCL (9.33 ± 0.94 cells/section) express Rbfox1 (Figure 3B).

One subclass of GABAergic ACs is defined by its expression of NPY. NPY-expressing cells in the mouse retina lie almost exclusively in the GCL and the innermost row of the INL [44]. Ramification of NPY cells in the IPL indicate that those located in the INL are likely involved in the OFF pathway, whereas those in the GCL are involved in the ON pathway. NPY cells have been shown to mediate RGCs tuning to low spatial frequencies; when NPY cells were selective ablated, ON- and OFF- RGCs that are tuned to low spatial frequencies lost this preferential tuning [45]. We observed an extensive overlap of Rbfox1 expression with NPY (Figure 3C). Most NPY cells in the GCL (6 ± 0.82 cells/section) and INL (17 ± 1.63 cells/section) were immunostained for Rbfox1; approximately 33 and 23% of NPY-positive cells in the GCL and INL, respectively, had very faint staining for Rbfox1 (Figure 3C).

In summary, Rbfox1 is expressed in both GABAergic and glycinergic ACs. The vast majority of Rbfox1-positive ACs were GABAergic. Within this type of AC, we showed expression of Rbfox1 in all starburst ACs and in most, if not all, NPY cells. Rbfox1 expression in glycinergic AC was very sparsely distributed and localized to VGlut3-expressing cells. Differential expression of Rbfox1 in different ACs, suggest its cell-type specific role in regulation of target RNA metabolism. One of the well-recognized and important functions of Rbfox1 is the regulation of genes that are involved in establishing neuronal circuits and synaptic transmission. Among those that have been shown to be differentially regulated in retinas of animals with Rbfox1 deletion were Vamp1 (vesicle-associated membrane protein-1), Vamp2, Snap25 (synaptosomal-associated protein 25), Trak2 (trafficking kinesin protein 2), and Slc1A7 (solute carrier family 1 member 7) [11]. Vamp1, Vamp2, and Snap25 together with syntaxins and synaptotagmin form the core of the SNARE [soluble *N*-ethylmaleimide-sensitive factor (NSF) attachment protein (SNAP) receptors] complex that mediates synaptic vesicle fusion [46]. Vamp1, in particular, has also been identified as a major Rbfox1 target in hippocampal neurons [47]. In the retina, however, Vamp1 immunostaining was restricted to a very few RGC somas and their dendrites, whereas abundant Vamp2 expression was observed in the IPL (contains synaptic connections between RGCs, ACs, and bipolar cells) and OPL (contains synaptic connections between photoreceptors, bipolar, and horizontal cells) [11,48]. This suggests that Vamp2 may play a major role in facilitating synaptic communications between retinal cells and, particularly between ACs and RGCs. Taken together, the data presented here contribute to our understanding about molecular differences between various types of AC and the cell-specific gene networks regulated by Rbfox1.

## Data Availability

The original data presented in the manuscript are available upon request.

## Abbreviations

AC: amacrine cell
CB: calbindin-D28K
ChAT: choline acetyltransferase
CM: calmodulin
CR: calretinin
DAPI: 4′,6-diamidino-2-phenylindole
GABA: gamma-aminobutyric acid
GCL: ganglion cell layer
GlyT1: glycine transporter
INL: inner nuclear layer
IPL: inner plexiform layer
NPY: neuropeptide Y
NSF: N-ethylmaleimide-sensitive factor
OPL: outer plexiform layer
PBS: phosphate buffered saline
PV: parvalbumin
RGC: retinal ganglion cells
RRM: RNA recognition motif
Snap25: synaptosomal-associated protein 25
SNARE: soluble N-ethylmaleimide-sensitive factor (NSF) attachment protein (SNAP) receptor
Vamp1: vesicle-associated membrane protein-1
VGlut3: vesicular glutamate transporter 3
